# The impact of parental socioeconomic characteristics and engagement on children’s early academic abilities: the role of racial and economic disparities

**DOI:** 10.3389/fpsyg.2026.1723683

**Published:** 2026-02-17

**Authors:** Cassandra Bolar, Nina Smith, Arthi Rao, Andrew Cooper, Gbemisola Talabi, Latrice Rollins, Brian McGregor

**Affiliations:** 1 School of Social Sciences, University of West Georgia, Carrollton, GA, United States; 2 College of Health and Sciences, North Carolina Central University, Durham, NC, United States; 3 Center for Health and Humanitarian Systems, Georgia Institute of Technology, Atlanta, GA, United States; 4 Department of Community Health and Preventive Medicine, Morehouse School of Medicine, Atlanta, GA, United States

**Keywords:** early childhood education, educational inequities, kindergarten, parental engagement, racial disparities, school readiness, SES

## Abstract

The current study utilized the Early Childhood Longitudinal Study to examine the impact of socioeconomic and parental engagement in the school system on children’s proficiency in math and reading in kindergarten. The analytic sample included 18,174 kindergarten children (47% White, 13% African American, 25% Hispanic, 9% Asian, 6% other; 51% male), and OLS regression revealed that the full models for math and reading were statistically significant explaining 22 and 16% of the variance in each outcome, respectively. Parental education, household income, and school-based parental involvement emerged as the strongest and most consistent predictors of children’s academic readiness, while marital status and parental depression were nonsignificant. Race/ethnicity and gender were also significant predictors, and African American and Hispanic students scored lower in math when compared to White students, whereas Asian children scored significantly higher than White peers in both math and reading. There was no statistically significant difference for reading scores between African American and White children. Girls demonstrated higher levels of reading readiness, and boys outperformed girls in math. Findings highlight the enduring influence of socioeconomic resources and parental engagement on early academic outcomes and underscore the need for policies and interventions that address structural inequities while promoting culturally responsive, family-centered support for early learning.

## 1 Introduction

Early childhood education establishes the foundation for children’s academic and life trajectories (Bakken et al., 2017; Ulferts et al., 2019). A quality education can serve as an important protective factor for reducing racial, economic, and intersectional inequities that drive disparate outcomes for racial minority children residing in households with limited resources (Browne and Battle, 2018; Coley et al., 2024; Njoroge and Omondi, 2024). Development and learning are occurring rapidly from birth to five, and no other timeframe in child development compares to this critical period of expedited brain growth and cognitive development (Gale et al., 2004; Gilmore et al., 2018; Prado and Dewey, 2014). Upon entry into kindergarten, children’s school readiness has been heavily shaped by their early childhood educational experiences and direct input and educational support from their parents. Educational interventions that focus on improving cognitive development for young children, ages 0–5, have promising longitudinal outcomes, such as improved literacy, kindergarten/academic readiness, academic achievement, graduation rates, and overall success in life (Barnett, 1995; Camilli et al., 2010). Juxtaposed with the critical impact of the educational system, caregivers also play an important role in influencing children’s educational outcomes, namely their kindergarten academic readiness.

The home environment is the first immediate context young children encounter, and caregivers’ parenting practices and parental engagement within the school system are heavily influenced by their own mental health and socioeconomic status (Borre and Kliewer, 2014; Hoff and Laursen, 2019; Posey-Maddox and Haley-Lock, 2020). Based on the central role of parental engagement on children’s early academic abilities, caregiver characteristics and parental involvement will be the focal predictors for the current study.

### 1.1 Theoretical framework

Rapid developmental changes in children’s cognitive, social, emotional, and moral capacities are occurring in early childhood, which have important implications for future learning and life outcomes (Bakken et al., 2017; Gilmore et al., 2018; Yoshikawa et al., 2020). The centrality of early learning experiences has long been emphasized by classic developmental theories. For example, Piaget highlighted the role of sensorimotor exploration in the development of early cognition and learning (Piaget, 1952). Vygotsky emphasized the sociocultural nature of learning that occurs through assisted learning and guided participation within the zone of proximal development (Vygotsky, 1978); and Bronfenbrenner conceptualized development as the result of dynamic interactions between the child and multiple ecological systems (Bronfenbrenner, 1979, 2000). Recent developmental research continues to affirm these foundational ideas, demonstrating that during early childhood, high-quality relational, linguistic, and learning environments contribute substantially to children’s early academic abilities (Powers et al., 2020; Rollins and Grindal, 2023).

Modern developmental theories further extend these ideas by highlighting the moral, cultural, and structural dimensions of development. Contemporary perspectives on sociocultural development and developmental systems theory view early achievement not only as a product of individual or family characteristics but also as shaped by structural inequities, racialized contexts, and culturally patterned parenting practices (Blackson et al., 2022; Garcia Coll and Marks, 2020). For example, sociocultural and cultural–ecological theories emphasize that parental beliefs and behaviors emerge within broader historical and community conditions that shape opportunity structures for racially minoritized children. Likewise, modern moral development scholarship highlights the ways inequitable environments influence children’s developing sense of fairness, belonging, and agency—inequities that are often visible as early as kindergarten entry (Rizzo and Killen, 2020; Elenbaas et al., 2020).

Despite these advances, few studies integrate both classical developmental theory and modern sociocultural perspectives to examine how parental socioeconomic characteristics and engagement shape early academic abilities within the context of racial disparities. Understanding these relationships is essential, given that racial/ethnic gaps in math and reading are already apparent at kindergarten entry and may reflect the cumulative effects of early home experiences, structural inequity, and differential access to developmental resources (Magnuson and Duncan, 2014; Reardon et al., 2023).

The current study aims to bridge these conceptual frameworks by examining how parental education, household income, parental involvement, and parental mental health predict kindergarten math and reading readiness within a racially diverse, nationally representative sample. Guided by Bronfenbrenner’s (2000) ecological theory and Belsky’s (1984) process model of parenting that examines how relational and individual factors influence parenting; and informed by modern sociocultural theories of race, culture, and opportunity, this study seeks to clarify how proximal parenting processes and distal structural forces jointly contribute to early academic disparities. In doing so, this work advances the developmental literature by integrating classical and contemporary theoretical perspectives to better understand the origins of inequality in early childhood learning.

### 1.2 School readiness

Broadly defined, school readiness refers to developmental indicators of preparedness such as cognitive, behavioral, emotional, social, and physical abilities. Prior studies consistently emphasize the importance of these markers in laying the foundation for children’s long-term success (Williams et al., 2019). School readiness may be especially important for Black children (Sawhill and Welch, 2023). For the purposes of the current study, the socio-emotional elements of school readiness, such as self-control, externalizing behaviors, and internalizing behaviors, were not utilized due to the limited variability of these outcomes within the current sample. Therefore, the remainder of the study focuses solely on early academic abilities as evidenced by kindergarten reading and math capabilities.

Historically, racial/ethnic achievement gaps have set Black children at a disadvantage for learning outcomes. Additionally, income disparities further heighten such gaps. By kindergarten entry, Black children typically trail their White peers by approximately nine months in math and seven months in reading (Reardon and Portilla, 2016; García and Weiss, 2017). These disparities do not stem not from innate ability but from unequal access to high-quality early learning environments, disparate neighborhood conditions, and inequitable economic resources in early childhood (Magnuson and Duncan, 2016; Yoshikawa et al., 2020).

Recent quasi-experimental and meta-analytic studies demonstrate that one year of high-quality pre-kindergarten or center-based childcare can substantially reduce early academic disparities, nearly eliminating the reading gap and cutting the math gap by as much as half at school entry (Weiland and Yoshikawa, 2013; Yoshikawa et al., 2013; Ansari et al., 2021). Furthermore, contemporary analyses demonstrate that a significant proportion of the Black–White test score gap at kindergarten entry can be explained by socioeconomic factors, including parental education, household income, residential stability, and access to early educational enrichment (Reardon, 2012; Reardon and Portilla, 2016; Coley et al., 2024; Reardon et al., 2023). However, SES and early environment don’t fully account for widening gaps during kindergarten. For instance, Reardon’s studies reveal that even when Black and White children enter the same school with similar readiness, Black children learn less over the kindergarten year—suggesting additional influences like differential treatment, bias, or disparities in schooling quality.

Recent studies have shed light on the origins of socio-cognitive processes which may underly both racial and gender biases. Findings from Geraci et al. (2024, 2025) reinforced the notion that intergroup attitudes, racial concepts, and gender biases emerge in childhood. Although the setting in which Geraci and colleagues explored these concepts falls outside of the United States educational context, authors investigating comparable concepts within the U.S. have unearthed similar patterns (Blackson et al., 2022). While early childhood educators are in a position to debunk such biases, research suggest that preschool teachers hold higher expectations of White students than Black students. Examples include white students receiving preferential learning opportunities and positive interactions with administration, as well as an overrepresentation of young Black children in special education and suspensions. Biases are detrimental to early educational experiences, readiness levels and overall wellbeing and warrant further exploration.

Although young children in early care and education settings spend a considerable amount of time with teachers, parents can play a critical role in shaping their preparedness for kindergarten. Scholars have emphasized several mechanisms through which early learning and developmental gains occur, including parental engagement, parenting-focused interventions, and parental beliefs about children’s learning and development (Sheridan et al., 2019; Wang and Sheikh-Khalil, 2014). Parental engagement has been consistently linked to young children’s social and emotional competencies, including self-regulation, empathy, and prosocial behavior (Dearing et al., 2015; Sheridan et al., 2019). Parent–child interactions characterized by warmth, sensitivity, emotional support, and active learning participation have been associated with improvements in attachment security, initiative, and other interpersonal abilities that support successful school adjustment (Bernier et al., 2016; Raby et al., 2015; Spinrad et al., 2020).

Interventions aimed at supporting parents have demonstrated added value beyond regular early care and education programming. Programs that build partnerships between ECE settings and parents often include home visits, workshops, and tailored supports—all aimed at reducing developmental vulnerabilities among children entering kindergarten. An additional line of inquiry that has arisen from intervention studies includes parental beliefs about what it means for a child to be school-ready. Some parents emphasize the importance of academic skills, while others have prioritized social and emotional abilities. In fact, such beliefs shape how much and in what ways parents engage in learning-supporting practices. In a study among Black and Hispanic families, mothers who placed a higher value on academic readiness engaged more in home literacy practices. The authors found that these practices mediated both reading and math achievement (Brinkley et al., 2022). Another large-scale study utilizing the Early Childhood Longitudinal Study-Kindergarten Cohort showed that both general beliefs about school readiness and beliefs about a particular child’s academic/behavioral competency predict different kinds of parental involvement which, in turn, are related to children’s academic outcomes. Examples of these types of parental engagement captured include school-based, classroom-based and homework help (Boyle and Benner, 2020). A common theme among studies is that parental beliefs and involvement differ by socioeconomic status and culture. Taken together, these findings emphasize the importance of further assessing the role of parental engagement in shaping young children’s early academic abilities. Additionally, demographic differences may be important factors to consider in interpreting findings.

Parenting practices and parental engagement within the school system are heavily influenced by the emotional and mental wellbeing of parents (Borre and Kliewer, 2014). For example, depression and anxiety have been related to lower levels of parental engagement in caretaking activities, educational activities with their children, and engagement within the school system as a whole (Rao et al., 2021). Furthermore, stress related to limited financial resources has resulted in harsher parenting styles and reduced parenting efficacy (Jackson and Choi, 2018; Simons et al., 2020). Considering the unique intersectionality of many minority parents with limited resources, their ability to stay positively engaged with their children and the school system may be compromised due to the unique environmental and psychological stressors that may be inherent in their lived experiences (Berryhill, 2016). Given the substantive empirical evidence that points to the critical role of parental engagement in the school system and its impact on children’s kindergarten academic readiness (Barnett et al., 2020), the current study aims to examine this relationship with keen attention given to the unique experiences of minority parents, varying SES contexts, and the impact of depression on children’s academic readiness in kindergarten.

### 1.3 Current study

As previously mentioned in the theoretical framework, the current study utilizes ecological theory (Bronfenbrenner, 2000) and Belsky’s (1984) process model for the determinants of parenting to understand the unique role that caregiver involvement and wellbeing (parental engagement, mental health, and socioeconomic status) play in children’s early academic abilities. More specifically, Belsky’s (1984) process model for parenting emphasizes the importance of personality and mental health in predicting parenting strategies, and ecological theory (Bronfenbrenner, 2000) emphasizes the importance of the environmental context in which child development and learning occur. The following is the proposed research question for the study:

1. How do caregiver characteristics (parental depression and socioeconomic status) and involvement (parental engagement in educational support) impact children’s early academic abilities as evidenced by their reading and math capabilities?

## 2 Materials and methods

### 2.1 Participants

Data from the Early Childhood Longitudinal Study (ECLS: K-2011) conducted by the National Center for Educational Statistics were utilized. This is a nationally representative sample of 20,000 kindergarten children from 1,352 public and private schools who started kindergarten during the 2010–2011 academic year, and they were assessed longitudinally through the fifth grade. The analytic sample for the current study included complete data for the chosen variables of analysis on 18,174 children during the fall semester of their kindergarten academic year. Fifty-one percent of the sample was male, and 49% of the sample was female. There was a diverse distribution of racial and ethnic backgrounds of students: 47% White, 13% African American, 25% Hispanic, 9% Asian, and 6% other. Data was collected from children, their families, teachers, and schools. Overall, the ECLS study focuses on children’s elementary school experiences, academic performance, and cognitive and socioemotional development.

### 2.2 Measures

#### 2.2.1 Math scores

Teacher reports of math scores were assessed during the fall semester of kindergarten. Teachers reported on students’ level of proficiency in mathematical thinking, which was one of the components of the larger Academic Rating Scale utilized for the study. Example items are the following: shows an understanding of the relationship between quantities, solves problems involving numbers using concrete objects, and orders a group of objects. There were eight items utilized to determine math scores. Responses for level of proficiency ranged from not yet, beginning, in progress, intermediate, to proficient. Scaled scores ranging from 0 to 100 were created for the final math score. Scaled scores were included to ensure that the two outcome variables were on similar scales from 0 to 100. Math scores and reading scores were on slightly different scales, and the scaled scores were used to make them comparable. Also, when using the log of the outcome to address mild left skewedness, the fit of the model did not vastly improve. Therefore, the scaled scores were utilized instead of the log transformed scores.

#### 2.2.2 Reading scores

Teacher reports of reading/literacy scores were assessed during the fall semester of kindergarten. The language and literacy section of the Academic Rating Scale was utilized. Children’s reading scores were calculated based on teacher responses to nine survey questions. The questions assessed whether the child could use complex sentence structures, comprehend a story read aloud, easily name all letters of the alphabet, predict subsequent events in stories, read simple books independently, apply strategies to read unfamiliar words, demonstrate early writing behaviors, compose simple stories, and exhibit an understanding of basic print conventions. Teachers rated each student’s ability using the categories not yet, beginning, in progress, intermediate, or proficient. Scaled scores ranging from 0 to 100 were created for the final reading score. Special note: Both the reading and math scores are rudimentary measures and serve as proxies and early potential indicators of the pre-academic skills that children need for success in school and life.

Parental Depression was assessed by a single item: How often during the past week have you felt depressed? Responses ranged from 1 (never), 2 (some of the time), 3 (moderate amount of the time), and 4 (most of the time).

Parental involvement within the school system was assessed by teachers’ responses to a single item: How involved at the school would you say this child’s parents/guardians are? Responses ranged from: 1: not involved at all, 2 (somewhat involved), 3 (very involved), and 4 (don’t know). Only 2.3% of the responses were 4 (don’t know), and this was for instances when the teacher was unaware of how involved caregivers were in their child’s school. This variable was treated as a nominal variable in analysis due to not knowing the parents’ involvement being rated as a 4 for this item. Based on best practices, missing data is not equivalent to not knowing a specific response.

Household income had 18 categories, which were the following: 1: $5K or less; 2: $5,001-$10K; 3: $10,001-$15K; 4: $15,001-$20K; 5: $20,001-$25K; 6: $25,001-$30K; 7: $30,001-$35K; 8: $35,001-$40K; 9: $40,001-$45K; 10: $45,001-$50K; 11: $50,001-$55K; 12: $55,001-$60K; 13: $60,001-$65K; 14: $65,001-$70K; 15: $70,001-$75K; 16: $75,001-$100K; 17: $100,001-$200K; 18: $200,001 or more.

Parental education for the primary caregiver was assessed as the following: 0: none, 1: 8th grade or below; 2: 9TH-12TH grade; 3: high school diploma/equivalent; 4: voc/tech program; 5: some college; 6: bachelor’s degree; 7: graduate/professional school—no degree; 8: master’s degree (MA, MS) or higher.

Marital status of primary caregiver was assessed as the following: 1: married, 2: separated, 3: divorced or widowed, 4: never married, 5: civil union/domestic partnership

Child race was assessed as the following: 1: White, non-Hispanic; 2: Black/African American, non-Hispanic; 3: Hispanic, race specified; 4: Hispanic, no race specified; 5: Asian, non-hispanic, 6: Others (Native Hawaiian/Pacific Islander (non-hispanic), American Indian/Alaska Native (non-hispanic), and two or more races (non-hispanic).

Child Sex: 1: male, 0: female.

### 2.3 Analytic strategy

Ordinary Least Squares Regression was utilized to estimate the impact of caregiver characteristics (parental depression and socioeconomic status) and involvement (parental engagement in educational support) on children’s kindergarten readiness as evidenced by their reading and math capabilities. Due to a slight skew in the distribution of the outcome variables, scaled scores were created for the math and reading scores that ranged from 0 to 100. Each academic outcome was examined separately. All statistical analyses were performed using JASP (2025) version 0.95.3, and the level of significance was set at 0.05.

## 3 Results

### 3.1 Math scores

The full OLS regression math model examining the impact of race, child sex, parental education, household income, marital status, parental involvement, and parental depression was statistically significant, *F*(40, 9243) = 65.25, *p* < 0.001, accounting for 22.0 percent of the variance of the scaled math scores (*R*^2^ = 0.220, Adjusted *R*^2^ = 0.217). The Durbin–Watson value (2.03) showed no autocorrelation, and all variance inflation factors VIF < 1.1, with no multicollinearity considerations (see Tables 1, 2).

**TABLE 1:** Math model summary.

Model	*R*	*R* ^2^	Adjusted *R*^2^	RMSE	AIC	BIC	*R*^2^ change	df1	df2	*p*	Durbin-Watson		
											Autocorrelation	statistic	*p*
M0	0.000	0.000	0.000	9.797	68723.146	68737.418	0.000	0	9283		–0.008	2.016	0.455
M1	0.469	0.220	0.217	8.670	66494.084	66793.798	0.220	40	9243	< 0.001	–0.015	2.029	0.159

M1 includes race, sex, parent edu, income, marital status, parent involvement, and depression.

**TABLE 2:** Math model ANOVA.

Model	Analysis	Sum of squares	df	Mean square	*F*	*p*
M1	Regression	196,188	40	4904.70	65.25	<0.001
	Residual	694,777	9243	75.17		
	Total	890,965	9283			

M1 includes race, sex, parent edu, income, marital status, parent involvement, and depression.

Parental education, household income, and parental involvement emerged as the strongest predictors of math scores. When compared to parents who reported no formal education, children of parents with a master’s degree or higher scored approximately β = 7.27 points higher (β = 7.27, *p* < 0.001). Children with parents who had a household income of $200,000 or higher scored about 5.33 points higher (*p* < 0.001) than children whose parents reported a household income of $5,000 or less. Parental involvement was also a significant positive predictor (β = 2.90, *p* < 0.001), indicating that higher levels of engagement were associated with better math performance. In contrast, marital status and parental depression were not significant predictors after accounting for other factors (see Table 3).

**TABLE 3:** Math OLS regression model summary.

Model	Predictors	Unstandardized	Standard error	Standardized^a^	t	*p*	95% CI		Collinearity statistics	
							Lower	Upper	Tolerance	VIF
M0	(Intercept)	20.666	0.102		203.249	<0.001	20.466	20.865		
M1	(Intercept)	12.317	0.890		13.842	<0.001	10.573	14.062		
Race	African American	−2.158	0.327		−6.591	<0.001	−2.800	−1.516	0.957	1.045
	Hispanic	−2.403	0.260		−9.225	<0.001	−2.914	−1.892		
	Hispanic (race not specified)	−2.815	1.745		−1.613	0.107	−6.236	0.607		
	Asian	2.274	0.393		5.782	<0.001	1.503	3.044		
	Others	0.450	0.381		1.180	0.238	−0.297	1.197		
Sex	Male	0.656	0.180		3.639	<0.001	0.303	1.010	0.998	1.002
Edu	9th–12th grade edu	−0.064	0.745		−0.086	0.931	−1.525	1.396	0.943	1.060
	HS diploma/equivalent	1.576	0.675		2.334	0.020	0.253	2.900		
	Vocational/tech program	3.105	0.763		4.070	<0.001	1.610	4.601		
	Some college	3.316	0.682		4.864	<0.001	1.979	4.652		
	Bachelor’s degree	5.435	0.705		7.710	<0.001	4.053	6.817		
	Grad/Prof. no degree	7.719	0.928		8.318	<0.001	5.900	9.538		
	Master’s degree and higher	7.267	0.720		10.095	<0.001	5.856	8.679		
Income	$5,001–$10,000	1.466	0.724		2.026	0.043	0.048	2.884	0.974	1.027
	$10,001–$15,000	1.713	0.671		2.554	0.011	0.399	3.028		
	$15,001–$20,000	1.044	0.664		1.573	0.116	−0.257	2.345		
	$20,001–$25,000	1.784	0.644		2.771	0.006	0.522	3.046		
	$25,001–$30,000	1.583	0.701		2.260	0.024	0.210	2.957		
	$30,001–$35,000	1.776	0.702		2.529	0.011	0.399	3.153		
	$35,001–$40,000	1.927	0.704		2.736	0.006	0.546	3.307		
	$40,001–$45,000	2.644	0.759		3.482	< 0.001	1.155	4.132		
	$45,001–$50,000	3.204	0.739		4.333	< 0.001	1.754	4.653		
	$50,001–$55,000	2.756	0.763		3.611	< 0.001	1.260	4.252		
	$55,001–$60,000	3.021	0.768		3.934	< 0.001	1.516	4.526		
	$60,001–$65,000	3.333	0.767		4.343	< 0.001	1.828	4.837		
	$65,001–$70,000	3.461	0.773		4.476	< 0.001	1.945	4.977		
	$70,001–$75,000	3.903	0.744		5.250	< 0.001	2.446	5.361		
	$75,001–$100,000	4.059	0.646		6.282	< 0.001	2.792	5.325		
	$100,001–$200,000	4.720	0.647		7.301	< 0.001	3.453	5.987		
	$200,000 or more	5.331	0.745		7.157	< 0.001	3.871	6.791		
Status	Separated	−0.801	0.482		−1.663	0.096	−1.746	0.143	0.946	1.057
	Divorced or Widowed	0.061	0.371		0.165	0.869	−0.666	0.788		
	Never Married	−0.210	0.317		−0.662	0.508	−0.831	0.411		
	Civil union/domestic Ptr	0.059	0.530		0.111	0.912	−0.981	1.099		
Involve	Somewhat involved	1.310	0.246		5.329	< 0.001	0.828	1.792	0.972	1.028
	Very involved	2.898	0.273		10.615	< 0.001	2.363	3.434		
	Did not know	1.218	0.661		1.843	0.065	−0.078	2.514		
Depress	Some of the time	−0.349	0.254		−1.371	0.170	−0.848	0.150	0.988	1.012
	Moderate amount of time	−0.518	0.579		−0.894	0.371	−1.654	0.618		
	Most of the time	−0.656	0.761		−0.862	0.389	−2.148	0.835		

Racial group differences in children’s math readiness were examined with White children serving as the reference category. After controlling for sex, parental education, income, marital status, parent involvement, and parental depression, several statistically significant racial differences emerged, *F*(40, 9243) = 65.25, *p* < 0.001. Results indicated that some racial and ethnic groups scored significantly higher or lower than White peers even after socioeconomic and family factors were taken into account (see Table 3).

African American children scored an average of β = 2.16 points lower than White children (β = -2.16, *p* < 0.001) on their math score. Similarly, Hispanic children (race specified) scored approximately β = 2.40 points lower (β = -2.40, *p* < 0.001). In contrast, Asian children scored significantly higher than White children, with an average advantage of β = 2.27 points (*p* < 0.001). Differences for Hispanic children with no race specified (β = –2.82, *p* = 0.107) and for those in the Other category—which included Native Hawaiian/Pacific Islander, American Indian/Alaska Native, and multiracial (non-Hispanic) children (β = 0.45, *p* = 0.238)—were not statistically significant. The nonsignificance for these categories may reflect smaller sample sizes and the heterogeneity of experiences within these groups. Lastly, as it relates to gender, males scored modestly higher than females (β = 0.66, *p* < 0.001) on their math scores (see Table 3).

### 3 3 Reading scores

An ordinary least squares (OLS) regression was conducted to examine the extent to which demographic, socioeconomic, and family factors predicted children’s reading readiness. The full model, which included race, sex, parental education, income, marital status, parent involvement, and parental depression, was statistically significant, *F*(40, 9258) = 46.34, *p* < 0.001, and explained 16.7% of the variance in scaled reading scores (*R*^2^ = 0.167, Adjusted *R*^2^ = 0.163). The Durbin–Watson statistic (2.02) indicated no autocorrelation in the residuals, and all variance inflation factors (VIFs < 1.1) suggested no multicollinearity concerns (see Tables 4, 5).

**TABLE 4:** Reading model summary.

Model	*R*	*R* ^2^	Adjusted *R*^2^	RMSE	AIC	BIC	*R*^2^ change	F^2^ change	df1	df2	*p*	Durbin-Watson		
												Autocorrelation	statistic	*p*
M0	0.000	0.000	0.000	9.098	67458.031	67472.306	0.000		0	9298		−0.006	2.011	.590
M1	0.408	0.167	0.163	8.323	65841.082	66140.864	0.167	46.34	40	9258	<0.001	−0.008	2.016	.446

M1 includes race, sex, parent edu, income, marital status, parent involvement, and depression.

**TABLE 5:** Reading model ANOVA.

Model	Analysis	Sum of squares	df	Mean square	*F*	*p*
M1	Regression	128,381	40	3209.52	46.34	<0.001
	Residual	641,266	9258	69.27		
	Total	769,647	9298			

M1 includes race, sex, parent edu, income, marital status, parent involvement, and depression.

Parental education and household income emerged as significant positive predictors of reading readiness. When compared to children with parents who reported no formal education, those with parents with a master’s degree or higher scored approximately β = 6.4 points higher on reading readiness (*p* < 0.001). Similarly, children from the highest income brackets scored up to 3.4 points higher than those from the lowest income group (*p* < 0.001). When controlling for all else in the model, parental involvement was also a strong and consistent predictor; higher levels of involvement were associated with reading scores that were β = 1.15–2.81 points higher than those of less-involved parents (*p* < 0.01) (see Table 6).

**TABLE 6:** Reading OLS regression model summary.

Model	Predictors	Unstandardized	Standard error	Standardized^a^	t	*p*	95% CI		Collinearity statistics	
							Lower	Upper	Tolerance	VIF
M0	(Intercept)	16.808	0.094		178.148	< 0.001	16.623	16.993		
M1	(Intercept)	10.501	0.853		12.318	< 0.001	8.830	12.172		
Race	African American	0.367	0.314		1.170	0.242	−0.248	0.983	0.958	1.044
	Hispanic	−0.984	0.250		−3.937	< 0.001	−1.474	−0.494		
	Hispanic (race not spec.)	−2.377	1.675		−1.419	0.156	−5.662	0.907		
	Asian	3.758	0.374		10.057	< 0.001	3.026	4.491		
	Other	0.994	0.365		2.723	0.006	0.278	1.709		
Sex	Male	−0.639	0.173		−3.693	< 0.001	−0.978	−0.300	0.998	1.002
Edu.	9–12th grade	−0.370	0.715		−0.516	0.606	−1.772	1.033	0.944	1.060
	High school diploma/equiv	1.058	0.648		1.633	0.103	−0.212	2.327		
	Vocational/tech program	2.082	0.732		2.842	0.004	0.646	3.517		
	Some college	2.567	0.653		3.928	< 0.001	1.286	3.848		
	Bachelor’s degree	4.224	0.675		6.254	< 0.001	2.900	5.548		
	Grad/Prof School No Deg	6.111	0.889		6.877	< 0.001	4.369	7.853		
	Master’s degree and higher	6.414	0.690		9.298	< 0.001	5.062	7.766		
Income	$5,000–$10,000	0.710	0.695		1.022	0.307	−0.652	2.071	0.974	1.027
	$10,001–$15,000	0.793	0.644		1.233	0.218	−0.468	2.055		
	$15,001–$20,000	0.457	0.636		0.718	0.473	−0.791	1.704		
	$20,001–$25,000	1.216	0.617		1.970	0.049	0.006	2.426		
	$25,001–$30,000	0.691	0.671		1.030	0.303	−0.624	2.006		
	$30,001–$35,000	1.281	0.673		1.904	0.057	−0.038	2.600		
	$35,001–$40,000	1.159	0.674		1.719	0.086	−0.163	2.481		
	$40,001–$45,000	1.768	0.727		2.431	0.015	0.343	3.194		
	$45,001–$50,000	2.327	0.708		3.286	0.001	0.939	3.714		
	$50,001–$55,000	1.744	0.732		2.383	0.017	0.310	3.179		
	$55,001–$60,000	2.096	0.736		2.849	0.004	0.654	3.538		
	$60,001–$65,000	1.639	0.736		2.228	0.026	0.197	3.081		
	$65,001–$70,000	2.276	0.741		3.071	0.002	0.823	3.730		
	$70,001–$75,000	2.535	0.712		3.562	< 0.001	1.140	3.930		
	$75,001–$100,000	2.825	0.619		4.565	< 0.001	1.612	4.038		
	$100,001–$200,000	2.749	0.619		4.440	< 0.001	1.536	3.963		
	$200,001 and Higher	3.424	0.714		4.797	< 0.001	2.025	4.824		
Status	Separated	−0.712	0.463		−1.539	0.124	−1.619	0.195	0.946	1.057
	Divorced or Widowed	−0.208	0.356		−0.584	0.559	−0.906	0.490		
	Never married	−0.253	0.304		−0.833	0.405	−0.849	0.342		
Civil union/domestic Ptr		−0.891	0.508		−1.753	0.080	−1.888	0.105		
Involve	Somewhat involved	1.153	0.236		4.890	< 0.001	0.691	1.615	0.972	1.028
	Very involved	2.811	0.262		10.733	< 0.001	2.298	3.325		
	Don’t know	1.851	0.633		2.924	0.003	0.610	3.091		
Depress	Some of the time	−0.138	0.244		−0.568	0.570	−0.617	0.340	0.988	1.012
	Moderate amount of time	−0.210	0.557		−0.377	0.706	−1.302	0.882		
	Most of the time	−0.903	0.730		−1.236	0.216	−2.335	0.529		

Racial group differences in reading readiness were examined with White children serving as the reference category. After controlling for sex, parental education, income, marital status, parental involvement, and parental depression, significant variation in reading readiness remained among racial and ethnic groups, *F*(5, 9258) = 46.34, *p* < 0.001. Hispanic children had reading scores that were one point lower on reading (β = –0.98, *p* < 0.001) than White children. By contrast, Asian children scored substantially higher than White children (β = 3.76, *p* < 0.001). Children classified as Other (including Native Hawaiian/Pacific Islander, American Indian/Alaska Native, and multiracial) scored slightly higher than White children (β = 0.99, *p* = 0.006), although this result should be interpreted with caution given the heterogeneity of this category. African American children did not differ significantly from White peers (β = 0.37, *p* = 0.242). Similarly, Hispanic children with no specified race showed a nonsignificant negative trend (β = –2.38, *p* = 0.156) (see Table 6).

Lastly, sex significantly predicted reading readiness, with boys scoring β = 0.64 points lower than girls (*p* < 0.001). In contrast, marital status and parental depression were not significant predictors after controlling for all other variables (see Table 6).

## 4 Discussion

Kindergarten serves as the foundation of children’s formal education, and this starting point is highly predictive of later educational, occupational, and life outcomes (Duncan and Magnuson, 2011; Entwisle et al., 2005). Disparate outcomes in early academic capabilities among minority children often lead to widening achievement gaps throughout their academic trajectories (Reardon and Portilla, 2016; Magnuson and Duncan, 2016). Racial and environmental inequities, including disparities in access to high-quality early childhood programs, neighborhood resources, and exposure to enriching home learning environments, are well-documented systemic drivers of these educational differences (García and Weiss, 2017; Bodovski, 2010; Yoshikawa et al., 2012). Despite the influence of these structural factors, parents play a pivotal role in shaping early educational outcomes through their socioeconomic resources, expectations, and direct engagement in children’s learning activities (Hart and Risley, 1995; Sénéchal and LeFevre, 2014; Wang et al., 2024).

The present study sought to understand how parents’ socioeconomic characteristics and educational involvement predict their children’s early academic abilities, as reflected in early proficiency in math and reading. The analyses revealed similar patterns across both math and reading scores in that parental education, household income, and parental involvement emerged as the most consistent and robust predictors, while marital status and parental depression were nonsignificant after controlling for the other variables. Data from the model for math accounted for 22% of the variance in math scores and 16.7% of variance in reading scores, which suggest that parental engagement in their child’s school and socioeconomic wellbeing explain a large proportion of early academic gaps. Taken together, these findings suggest that early academic abilities are influenced by multiple factors such as economic, structural, and parental factors.

The present study contributes to longstanding developmental debates on the origins of early academic disparities by demonstrating how parental education, household income, and parental involvement (core components of both proximal processes and sociocultural context) jointly predict children’s math and reading readiness at kindergarten entry (Bradley and Corwyn, 2002; Davis-Kean, 2005; Reardon and Portilla, 2016; James-Brabham et al., 2023). The findings reinforce classical developmental theories while also extending modern developmental scholarship on sociocultural inequities and racially patterned opportunity structures that shape early learning opportunities (Blackson et al., 2022; García and Weiss, 2017; Yoshikawa et al., 2020).

From a classical perspective, the results are consistent with Bronfenbrenner’s ecological theory, which posits that children’s developmental outcomes arise from interactions between family-level processes and broader structural conditions (Bronfenbrenner, 2000). High parental education and income likely enhance the microsystem through enriched home learning opportunities, while parental involvement strengthens mesosystem connections between home and school (Davis-Kean, 2005; Barnett et al., 2020; Wang and Wei, 2024). Similarly, the findings align with Vygotskian theory: parents with greater educational and socioeconomic resources may provide more intentional scaffolding, richer language interactions, and more frequent learning supports, all of which facilitate the development of early cognitive skills (Sénéchal and LeFevre, 2014; Ramani et al., 2015; Nelson et al., 2024).

One of the most compelling findings of the study was in fact a non-significant finding: African American children’s reading scores were not statistically different from White children when controlling for the other variables in the study. There is research noting that Black and White educational gaps are lessening (Reardon and Portilla, 2016), and the current study highlights that there is no statistically significant difference between White and Black children’s early proficiency in reading in kindergarten. This finding leads to additional questions. Is this nonsignificant finding for reading between African American and White children persistent across the elementary school years? If this finding changes over time, how might school-related and parent-level variables predict changes in children’s literacy across the elementary school years. The ECLS dataset would lend itself well to examining these potential questions due to its longitudinal research design.

For both math and reading scores, parental education demonstrated the strongest influence on children’s readiness scores. Children whose parents attained the highest education level (a master’s degree or higher) scored over seven points higher in math and up to six points higher in reading than children of parents with less than an 8th grade education. These results corroborate a large body of evidence suggesting that parental education functions as a proxy for cognitive and cultural capital, shaping home learning environments and parenting practices that support early academic development (Martinez et al., 2022; Li et al., 2025). Parents with greater educational attainment typically provide more cognitively stimulating experiences, engage in higher-quality language and math interactions, and maintain consistent expectations regarding school success (Hart and Risley, 1995; Davis-Kean, 2005). Furthermore, higher parental education often translates into greater proficiency in navigating school systems, which may lead to increased advocacy and engagement in children’s learning (Blevins-Knabe and Musun-Miller, 1996).

Despite parental education, household income, and parental involvement explaining a considerable amount of variance in reading and math scores, racial disparities persisted in these outcomes. The influence of structural racism operating across various ecological levels may provide a possible explanation. Structural racism includes both historical and contemporary institutional practices, policies, and systems that differentially allocate resources, risks, and opportunities across racial groups; often these structural inequities play a critical role in influencing individual-level socioeconomic outcomes (Bailey et al., 2017; Gee and Hicken, 2021).

Early childhood exposure to inequities in neighborhood conditions, access to high-quality learning environments, school funding, and teacher expectations, which are disproportionately experienced by racial minority children, shape early learning opportunities prior to and during kindergarten entry (Blackson et al., 2022; Darling-Hammond, 2015). Despite holding parental SES and engagement constant, these broader structural conditions may constrain the translation of family resources into equivalent academic returns for Black and Hispanic children, which is a phenomenon consistent with theories of diminished returns under racial stratification (Assari, 2018).

Research has demonstrated that racial bias experienced in early childhood educational settings may contribute to inequities in teaching quality, discipline strategies, and expectations; especially in math-related domains in which stereotypes about ability are pervasive (Cimpian et al., 2016; Okonofua and Eberhardt, 2015). Such mechanisms may explain why disparities persisted in math scores and not reading scores for African American children in the current study. Taken together, the findings of the current study should be considered based on the understanding that early academic disparities are not solely the product of family-level characteristics but are embedded within broader systems of racialized opportunity.

Household income also demonstrated a strong, graded association with both math and reading readiness. Children from higher-income households scored between three and five points higher in math and up to 3.4 points higher in reading than peers from low-income households. These findings are consistent with extensive research linking socioeconomic status (SES) to early learning outcomes through both material and psychosocial pathways (Bradley and Corwyn, 2002; Ferguson et al., 2007; James-Brabham et al., 2023). Financially stable families can provide access to high-quality preschools, learning materials, and extracurricular opportunities that foster cognitive growth, whereas economic hardship can increase stress and reduce parents’ emotional availability and cognitive engagement with children (Yoshikawa et al., 2012). Notably, SES differences often translate into disparities in early math skills and literacy exposure long before kindergarten entry (Reardon, 2012), illustrating how income-based inequalities shape the foundation for future academic achievement.

Parental involvement emerged as a significant and independent predictor of both math and reading readiness, even after controlling for SES. Children whose parents demonstrated higher involvement in the school system scored between one and three points higher in their math and reading scores. This pattern aligns with meta-analytic findings that active parental engagement in rudimentary math and literacy activities—such as shared book reading, math talk, and guided play—enhances children’s cognitive development (Sénéchal and LeFevre, 2014; Wang et al., 2024). Involvement serves as both a promotive and compensatory mechanism: engaged parents help children from lower-income backgrounds overcome structural disadvantages by fostering home environments rich in learning opportunities (Nelson et al., 2024; Sheridan et al., 2019). Importantly, parental involvement is malleable and can be strengthened through interventions that train parents in language- and math-rich interactions (Mol et al., 2008).

For math scores, despite controlling for SES and family processes, racial disparities persisted in math readiness. African American and Hispanic children scored significantly lower than White peers by approximately 2.16 and 2.40 points, respectively, in math scores. These gaps are consistent with national data demonstrating persistent early academic disparities (Reardon and Portilla, 2016; Duncan and Magnuson, 2011). Such differences likely reflect systemic inequities in access to quality early education, exposure to academically enriched environments, and the cumulative effects of residential and economic segregation (Bodovski, 2010; Entwisle et al., 2005). For Hispanic children, additional factors such as language environment, bilingual development, and underrepresentation in formal early education programs contribute to initial readiness gaps (Magnuson et al., 2006; Mancilla-Martinez and Lesaux, 2011). However, bilingual children often display rapid growth once they enter supportive, language-inclusive environments, suggesting that these gaps are contextual rather than innate.

Conversely, Asian children scored substantially higher than White peers in both math (+2.27 points) and reading (+3.76 points), consistent with research documenting the “Asian achievement advantage” (Lee and Zhou, 2015). This pattern is often attributed to cultural values emphasizing effort, discipline, and educational success, as well as structured home learning environments that promote numeracy and literacy (Huntsinger et al., 2000; Cheung and Pomerantz, 2011). Such family-based practices highlight the importance of sociocultural context in shaping academic readiness. Children categorized as Other (Native Hawaiian/Pacific Islander, American Indian/Alaska Native, and multiracial, non-Hispanic) showed small, nonsignificant differences relative to White peers, though subgroup variation within this category may mask meaningful disparities (Quintana et al., 2012).

Sex differences also emerged across both outcomes: boys outperformed girls in math, while girls outperformed boys in reading. These findings mirror well-documented gendered patterns in early development, reflecting both biological maturation differences and sociocultural expectations (Logan and Johnston, 2010; Cimpian et al., 2016).

Overall, the findings highlight that early math and reading readiness are shaped by both structural resources and important parent socioeconomic characteristics and parenting processes, but are also influenced by enduring systemic inequities. Socioeconomic and parental engagement variables explain a substantial proportion of early academic variability, yet residual racial differences indicate the need for policies and interventions addressing structural inequities and promoting culturally responsive pedagogy. Early childhood programs that integrate family engagement training, equitable access to preschool, and linguistically inclusive instruction, such as *Reach Out and Read* or bilingual math talk initiatives, are critical for closing early readiness gaps (Ramani et al., 2019). Future research should examine longitudinally how family engagement mediates SES effects and whether culturally adapted interventions can sustain gains in both literacy and numeracy across diverse populations.

## 5 Limitations

The models utilized for analysis were constructed based on the largest sample size with the least amount of missing data. As a result, the models focus on parental characteristics and their association with learning outcomes. We acknowledge that a more complete narrative should ideally also include school-level characteristics since learning occurs both in the home and the school environment. We fully explored this using an iterative process. Our first step was to assess the nested patterns of students within schools. Our analysis revealed insufficient sample sizes at lower and upper levels to construct a robust two-level multilevel model. Our next step was to subset schools that contained only one student per school (*n* = 302). Our rationale was to still be able to run OLS models without violating their underlying assumptions of independence of observations. However, the data documentation suggests that these are schools with poor response rates, and they also had high rates of missingness across school characteristics (socioeconomic composition, resource/facility availability, student problems, teaching quality). Further subsetting the data to eliminate records with missing data yielded a sample with insufficient degrees of freedom to estimate coefficients for both parent-level and school-level predictors (*n* = 117). The models run with this smaller dataset yielded non-significant models, likely due to the sample size limitations. For future work, we aspire to explore and build a more robust sample that will enable us to include both parent-level and school-level characteristics and construct a more complete narrative, to test the hypothesis that parental characteristics have an independent impact on student outcomes after controlling for school characteristics.

Beyond the analytic limitations, the study’s cross-sectional design restricts causal inference. Although the findings identify significant associations between family variables and children’s early academic skills, they do not establish temporal or causal relationships. The current study was exploratory in nature and focused on the simple effects of predictors, which could have limited the understanding of the complex interplay that may exist between key predictor variables, especially the impact of race/ethnicity and SES on parental involvement in the school system and parents’ economic characteristics. Future analysis should examine the interaction between race/ethnicity and SES on the key predictors and the overall outcome variables.

In addition, reliance on parent-reported measures introduces the possibility of social desirability bias or underreporting, particularly regarding depression (Sénéchal and LeFevre, 2014). Additionally, depression was assessed by a single question (How often during the past week have you felt depressed?), in which there was little variability with 80% of respondents reporting that they never felt depressed within the past week. Furthermore, one week is a short retrospective timeframe to assess a person’s mental state. Finally, while racial categories were included, their broad aggregation may mask within-group variability across cultural, linguistic, and immigration-related experiences (Crosnoe et al., 2016).

In sum, although this study provides valuable insights into the role of parental socioeconomic characteristics and engagement in early math and reading readiness, it is limited by the exclusion of school-level data and the cross-sectional nature of the design. Future research should leverage multilevel longitudinal datasets to capture the complex, nested relationships among home, school, and community factors that shape early academic development.

## Data Availability

Publicly available datasets were analyzed in this study. This data can be found at: https://nces.ed.gov/ecls/dataproducts.asp.

## References

[B1] Ansari A. Pianta R. C. Whittaker J. E. Vitiello V. Ruzek E. (2021). Enrollment in public-prekindergarten and school readiness skills at kindergarten entry: Differential associations by home language, income, and program characteristics. *Early Child. Res. Q.* 54 60–71. 10.1016/j.ecresq.2020.07.011

[B2] Assari S. (2018). Unequal gain of equal resources across racial groups. *Int. J. Health Policy Manag.* 7 1–9. 10.15171/ijhpm.2017.90 29325397 PMC5745862

[B3] Bailey Z. D. Krieger N. Agénor M. Graves J. Linos N. Bassett M. T. (2017). Structural racism and health inequities in the USA: Evidence and interventions. *Lancet* 389 1453–1463. 10.1016/S0140-6736(17)30569-X 28402827

[B4] Bakken L. Brown N. Downing B. (2017). Early childhood education: The long-term benefits. *J. Res. Child. Educ.* 31 255–269. 10.1080/02568543.2016.1273285

[B5] Barnett M. A. Paschall K. W. Mastergeorge A. M. Cutshaw C. A. Warren S. M. (2020). Influences of parent engagement in early childhood education centers and the home on kindergarten school readiness. *Early Child. Res. Q.* 53 260–273. 10.1016/j.ecresq.2020.05.005

[B6] Barnett W. S. (1995). Long-term effects of early childhood programs on cognitive and school outcomes. *Future Child.* 5 25–50. 10.2307/1602366 8835514

[B7] Belsky J. (1984). The determinants of parenting: A process model. *Child Dev.* 55 83–96. 10.1111/j.1467-8624.1984.tb00275.x 6705636

[B8] Bernier A. Matte-Gagné C. Bélanger M.-E. Whipple N. (2016). Taking stock of two decades of attachment transmission gap: Broadening the assessment of maternal sensitivity. *Child Dev.* 87 271–288. 10.1111/cdev.12467 24611791

[B9] Berryhill M. B. (2016). Mothers’ parenting stress and engagement: Mediating role of parental competence. *Marriage Fam. Rev.* 52 461–480. 10.1080/01494929.2015.1113600

[B10] Blackson E. A. Gerdes M. Segan E. Anokam C. Johnson T. J. (2022). Racial bias toward children in the early childhood education setting. *J. Early Child. Res.* 20 277–292. 10.1177/1476718X221087051

[B11] Blevins-Knabe B. Musun-Miller L. (1996). Number use at home by children and their parents and its relationship to early mathematical performance. *Early Dev. Parent.* 5 35–45. 10.1002/(SICI)1099-0917(199603)5:1<35::AID-EDP113<3.0.CO;2-0

[B12] Bodovski K. (2010). Parental practices and educational achievement: Social class, race, and habitus. *Br. J. Sociol. Educ.* 31 139–156. 10.1080/01425690903539024

[B13] Borre A. Kliewer W. (2014). Parental strain, mental health problems, and parenting practices: A longitudinal study. *Pers. Individ. Dif.* 68 93–97. 10.1016/j.paid.2014.04.014 24976666 PMC4067046

[B14] Boyle A. E. Benner A. D. (2020). Understanding parental educational involvement: The roles of parental general and child-specific school readiness beliefs. *Merrill Palmer Q.* 66:4. 10.13110/merrpalmquar1982.66.2.0199 38347919 PMC10860271

[B15] Bradley R. H. Corwyn R. F. (2002). Socioeconomic status and child development. *Annu. Rev. Psychol.* 53 371–399. 10.1146/annurev.psych.53.100901.135233 11752490

[B16] Brinkley D. Y. Caughy M. O. Owen M. T. (2022). Mothers’ school readiness beliefs and literacy involvement and children’s academic outcomes in ethnic minority families. *Early Educ. Dev.* 34 1–16. 10.1080/10409289.2022.2089505 40727879 PMC12302957

[B17] Bronfenbrenner U. (1979). *The ecology of Human Development: Experiments by Nature and Design.* Cambridge, MA: Harvard University Press.

[B18] Bronfenbrenner U. (2000). *Ecological Systems Theory.* Washington, DC: American Psychological Association.

[B19] Browne A. P. Battle J. (2018). Black family structure and educational outcomes: The role of household structure and intersectionality. *J. Afr. Am. Stud.* 22 77–93. 10.1007/s12111-018-9395-7

[B20] Camilli G. Vargas S. Ryan S. Barnett W. S. (2010). Meta-analysis of the effects of early education interventions on cognitive and social development. *Teach. Coll. Rec.* 112 579–620. 10.1177/016146811011200303

[B21] Cheung C. S.-S. Pomerantz E. M. (2011). Parents’ involvement in children’s learning in the United States and China. *Child Dev.* 82 932–950. 10.1111/j.1467-8624.2011.01582.x 21418057 PMC3089668

[B22] Cimpian J. R. Lubienski S. T. Timmer J. D. Makowski M. B. Miller E. K. (2016). Have gender gaps in math closed? *AERA Open* 2:2332858416673617. 10.1177/2332858416673617

[B23] Coley R. L. Carey N. Hwang D. Spielvogel B. Henry D. (2024). Racial inequities in educational opportunity. *Race Soc. Probl.* 16 414–432. 10.1007/s12552-024-09415-z

[B24] Council on Early Childhood High P. C. Klass P. Donoghue E. Glassy D. DelConte B. (2014). Literacy promotion: An essential component of primary care pediatric practice. *Pediatrics* 134 404–409. 10.1542/peds.2014-1384 24962987

[B25] Crosnoe R. Ansari A. Purtell K. M. Wu N. (2016). Latin American immigrant parents and their children’s teachers in U.S. early childhood education programs. *Int. J. Psychol.* 51 753–762. 10.1002/ijop.12135 26010079 PMC4661132

[B26] Darling-Hammond L. (2015). *The Flat World and Education: How America’s Commitment to Equity will Determine our Future.* New York, NY: Teachers College Press.

[B27] Davis-Kean P. E. (2005). The influence of parent education and family income on child achievement. *J. Fam. Psychol.* 19 294–304. 10.1037/0893-3200.19.2.294 15982107

[B28] Dearing E. McCartney K. Taylor B. A. (2015). Within-child associations between family–school connections and social skills. *Child Dev.* 86 1338–1355. 10.1111/cdev.12390 26110500

[B29] Duncan G. J. Magnuson K. (2011). “The nature and impact of early achievement skills,” in *Whither Opportunity?*, eds Duncan G. J. Murnane R. J. (New York, NY: Russell Sage), 47–70.

[B30] Elenbaas L. Rizzo M. T. Cooley S. Killen M. (2020). Evaluations of intergroup exclusion. *Child Dev.* 91 87–104. 10.1111/cdev.13160 31144306 PMC9048094

[B31] Entwisle D. R. Alexander K. L. Olson L. S. (2005). First grade and educational attainment. *Am. J. Sociol.* 110 1458–1502. 10.1086/428444

[B32] Ferguson H. B. Bovaird S. Mueller M. P. (2007). Impact of poverty on educational outcomes. *Paediatr. Child Health* 12 701–706. 10.1093/pch/12.8.701 19030450 PMC2528798

[B33] Gale C. R. O’Callaghan F. J. Godfrey K. M. Law C. M. Martyn C. N. (2004). Critical periods of brain growth. *Brain* 127 321–329. 10.1093/brain/awh034 14645144

[B34] Garcia Coll C. Marks A. K. (2020). *The Immigrant Paradox in Children and Adolescents.* Washington, DC: American Psychological Association.

[B35] García E. Weiss E. (2017). *Education Inequalities at the School Starting Gate.* Washington, DC: Economic Policy Institute.

[B36] Gee G. C. Hicken M. T. (2021). Structural racism: The rules and relations of inequity. *Ethn. Dis.* 31(Suppl. 1), 293–300. 10.18865/ed.31.S1.293 34045831 PMC8143846

[B82] Geraci A. Commodari E. Perucchini P. (2024). Early intergroup coalition: Toddlers attribute fair distributions to Black rather than White distributors. *Soc. Dev*. 33:e12740. 10.1111/sode.12740

[B83] Geraci A. Perucchini P. Surian L. (2025). The development of gender biases in the attribution of distributive fairness. *European Journal of Developmental Psychology*. 22, 745–770. 10.1080/17405629.2025.2536851

[B37] Gilmore J. H. Knickmeyer R. C. Gao W. (2018). Imaging structural and functional brain development in early childhood. *Nat. Rev. Neurosci.* 19 123–137. 10.1038/nrn.2018.1 29449712 PMC5987539

[B38] Hart B. Risley T. R. (1995). *Meaningful Differences in the Everyday Experience of Young American Children.* Baltimore, MD: Paul H. Brookes.

[B39] Hoff E. Laursen B. (2019). “Socioeconomic status and parenting,” in *Handbook of Parenting*, ed. Bornstein M. H. (New York, NY: Routledge), 421–447.

[B40] Huntsinger C. S. Jose P. E. Larson S. L. Balsink Krieg D. Shaligram C. (2000). Academic development in Chinese American and European American children. *J. Educ. Psychol.* 92 745–760.

[B41] Jackson A. P. Choi J. (2018). Parenting stress and harsh parenting. *J. Fam. Med. Commun. Health* 5:10.

[B42] James-Brabham E. Loveridge T. Sella F. Wakeling P. Carroll D. J. Blakey E. (2023). How do Socioeconomic attainment gaps in early mathematical ability arise? *Child Dev.* 94 1550–1565. 10.1111/cdev.13947 37248732 PMC10953023

[B43] Lee J. Zhou M. (2015). *The Asian American Achievement Paradox.* New York, NY: Russell Sage Foundation.

[B44] Li Z. Chen K. Rosales K. P. Xu J. Looney L. Zhou X. (2025). Children’s math and vocabulary learning. *Behav. Sci.* 15:527. 10.3390/bs15040527 40282146 PMC12023977

[B45] Logan S. Johnston R. (2010). Gender differences in reading. *Educ. Rev.* 62 175–187.

[B46] Magnuson K. Duncan G. J. (2014). Early childhood interventions and inequality. *RSF J. Soc. Sci.* 2 123–141. 10.7758/RSF.2016.2.2.05 PMC6100797 30135867

[B47] Magnuson K. Duncan G. J. (2016). Can early childhood interventions decrease inequality of opportunity? *RSF J. Soc. Sci.* 2, 123–141. 10.7758/RSF.2016.2.2.05 PMC6100797 30135867

[B48] Magnuson K. Lahaie C. Waldfogel J. (2006). Preschool and school readiness of immigrant children. *Soc. Sci. Q.* 87, 1241–1262.

[B49] Mancilla-Martinez J. Lesaux N. K. (2011). Spanish speakers’ literacy gaps. *Child Dev.* 82 1544–1560. 21848955 10.1111/j.1467-8624.2011.01633.x PMC3169767

[B50] Martinez J. L. Hasty C. Morabito D. Maranges H. M. Schmidt N. B. Maner J. K. (2022). Childhood unpredictability and adult outcomes. *Dev. Psychopathol.* 34 705–717. 35039110 10.1017/S0954579421001607

[B84] Mol S. E. Bus A. G. De Jong M. T. Smeets D. J. (2008). Added value of dialogic parent-child book readings: A meta-analysis. *Early Educ. Dev*. 19, 7–26.

[B51] Nelson G. Carter H. Boedeker P. Knowles E. Buckmiller C. Eames J. (2024). Math interventions with caregivers. *Rev. Educ. Res.* 94 112–152. 10.3102/00346543231156182 38293548

[B85] Njoroge J. Omondi M. (2024). Unveiling the intersectionality of socioeconomic status, race, and educational attainment: A comprehensive analysis of equity and access in contemporary education systems. *Soc. Dyn. Rev*. 7, 1–8.

[B52] Okonofua J. A. Eberhardt J. L. (2015). Two strikes: Race and the disciplining of young students. *Psychol. Sci.* 26 617–624. 10.1177/0956797615570365 25854276

[B53] Piaget J. (1952). *The Origins of Intelligence in Children.* New York, NY: International Universities Press.

[B54] Posey-Maddox L. Haley-Lock A. (2020). Parent engagement across contexts. *Urban Educ.* 55 671–698. 10.1177/0042085916660348

[B55] Powers J. N. Farewell C. V. Maiurro E. Puma J. (2020). Workplace wellness in early childhood education. *Workplace Health Saf.* 68 65–72. 10.1177/2165079919882732 31752625

[B56] Prado E. L. Dewey K. G. (2014). Nutrition and brain development. *Nutr. Rev.* 72 267–284. 10.1111/nure.12102 24684384

[B57] Quintana S. M. Chew A. Shell G. (2012). “Counseling psychology and race,” in *APA Handbook of Counseling Psychology, Vol. 2: Practice, Interventions, and Applications*, eds Fouad N. A. Carter J. A. Subich L. M. (Washington, DC: American Psychological Association), 453–489. 10.1037/13754-020

[B58] Raby K. L. Roisman G. I. Fraley R. C. Simpson J. A. (2015). Early maternal sensitivity and adult competence. *Child Dev.* 86 695–708. 10.1111/cdev.12325 25521785 PMC4428971

[B59] Ramani G. B. Rowe M. L. Eason S. H. Leech K. A. (2015). Math talk in Head Start families. *Cogn. Dev.* 35 15–33.

[B60] Ramani S. Könings K. D. Ginsburg S. van der Vleuten C. P. M. (2019). Promoting feedback culture. *Med. Teach.* 41 625–631. 29411668 10.1080/0142159X.2018.1432850

[B61] Rao Z. Barker B. O’Farrelly C. Ramchandani P. (2021). Maternal mental health and pretend play. *BMC Psychol.* 9:70. 10.1186/s40359-021-00568-9 33957981 PMC8103647

[B62] Reardon S. F. (2012). The widening academic achievement gap between the rich and the poor: New evidence and possible explanations. *Commun. Investm.* 24 19–39.

[B63] Reardon S. F. Kalogrides D. Ho A. D. (2023). Trends in achievement gaps. *Educ. Res.* 52 143–156. 10.3102/0013189X231160288 38293548

[B64] Reardon S. F. Portilla X. A. (2016). School readiness gaps. *AERA Open* 2, 1–18. 10.1177/233285841665734326942210

[B65] Rizzo M. T. Killen M. (2020). Resource inequality origins. *Child Dev.* 91 1155–1168. 10.1111/cdev.13181 30370937

[B66] Rollins B. Y. Grindal T. (2023). Early childhood environments and readiness. *Annu. Rev. Dev. Psychol.* 5 123–148. 10.1146/annurev-devpsych-070220-120007

[B67] Sawhill I. V. Welch M. (2023). *Early Childhood Education: What Do We Know and What Should We Do?* Washington, DC: Brookings Institution.

[B68] Sénéchal M. LeFevre J.-A. (2014). Home literacy environment. *Child Dev.* 85 1552–1568. 10.1111/cdev.12222 24467656

[B69] Sheridan S. M. Smith T. E. Kim E. M. Beretvas S. N. Park S. (2019). Family–school interventions meta-analysis. *Rev. Educ. Res.* 89 296–332. 10.3102/0034654318825437 38293548

[B70] Simons R. L. Whitbeck L. B. Melby J. N. Wu C.-I. (2020). “Economic pressure and harsh parenting,” in *Families in Troubled Times* (New York, NY: Routledge), 207–222.

[B71] Spinrad T. L. Eisenberg N. Cumberland A. Valiente C. (2020). Emotion-related self-regulation. *Dev. Psychopathol.* 32 140–164.

[B72] Ulferts H. Wolf K. M. Anders Y. (2019). Process quality in ECEC. *Child Dev.* 90 1474–1489. 10.1111/cdev.13296 31407322

[B73] Vygotsky L. S. (1978). *Mind in Society.* Cambridge, MA: Harvard University Press.

[B74] Wang M.-T. Sheikh-Khalil S. (2014). Parental involvement. *Dev. Psychol.* 50 237–252. 10.1037/a0033479 23544854

[B75] Wang X. Chan K. K. Li Q. Leung S. O. (2024). Computational thinking meta-analysis. *J. Educ. Comput. Res.* 62 962–988.

[B76] Wang X. Wei Y. (2024). Parental involvement and math performance. *Front. Psychol.* 15:1463359. 10.3389/fpsyg.2024.1463359 39742039 PMC11686222

[B77] Weiland C. Yoshikawa H. (2013). Prekindergarten impacts. *Child Dev.* 84 2112–2130. 10.1111/cdev.12099 23534487

[B78] Williams P. G. Lerner M. A. Council on Early Childhood, Council on School Health, Sells J. Alderman S. L. (2019). School readiness. *Pediatrics* 144:e20191766. 31331984 10.1542/peds.2019-1766

[B79] Yoshikawa H. Aber J. L. Beardslee W. R. (2012). Poverty effects on child mental health. *Am. Psychol.* 67 272. 10.1037/a0028015 22583341

[B80] Yoshikawa H. Weiland C. Brooks-Gunn J. Burchinal M. R. Espinosa L. M. Gormley W. (2013). Investing in our future. *Soc. Policy Rep.* 27 1–40.

[B81] Yoshikawa H. Wuermli A. J. Britto P. R. Dreyer B. Leckman J. F. Lye S. J. (2020). COVID-19 and early childhood development. *Lancet Child Adolesc. Health* 4 495–501. 10.1016/S2352-4642(20)30106-532497520

